# Parental health literacy and multidimensional health education competencies in preschool children

**DOI:** 10.3389/fpubh.2026.1984037

**Published:** 2026-10-29

**Authors:** Hasan Uğur Serdaroğlu, Gonca Kurt

**Affiliations:** 1 Department of Child Care and Youth Services, Pazar Vocational School of Higher Education, Tokat Gaziosmanpaşa University, Tokat, Türkiye; 2 Department of Health Care Services, Pazar Vocational School of Higher Education, Tokat Gaziosmanpaşa University, Tokat, Türkiye

**Keywords:** early childhood, health education, health literacy, health promotion, parental health literacy, preschool children

## Abstract

**Objective:**

Although parental health literacy is regarded as correlated with the health-related learning environment of preschool children, evidence addressing children’s health education in a multidimensional manner remains limited. Thus, the aim of this study was to examine the relationship between parental health literacy and the parent-reported overall health education score and its subdimensions among preschool children—after controlling for child-, parent-, and family-related characteristics—as well as the incremental explanatory contribution of parental health literacy beyond these characteristics.

**Method:**

This cross-sectional study was conducted in 2024 with 407 parents of 5 and 6-year-old children enrolled in preschools in Tokat, Türkiye. Data were collected using the Health Literacy Scale—Short Form, the Parent-Reported Health Education Scale for Preschool Children, and the Sociodemographic and Health Information Form. Child-, parent-, and family-related characteristics, as well as parental health literacy, were included in hierarchical multiple linear regression models, respectively. The overall health education score and its six subscales were analyzed separately. The Benjamini-Hochberg correction was applied to the *p*-values for parental health literacy in the subscale models.

**Results:**

The final model explained 39.5% of the variance in the overall health education score. Including parental health literacy in the model contributed an additional 4.1% to the explained variance (Δ*R*^2^ = 0.041; *p* < 0.001), and a higher level of parental health literacy correlated with higher overall scores in health education (*B* = 0.012; *β* = 0.215; 95% CI: 0.008–0.017). Following the Benjamini-Hochberg correction, parental health literacy remained significantly associated with higher scores on the subscales of personal safety and first aid, hygiene and self-care, nutrition, mental health and social relationships, and neglect and abuse; however, no significant relationship was found for the sleep subscale.

**Conclusion:**

Higher parental health literacy was found to be linked with higher overall health education scores among preschool children and higher scores in five of the six domains, after controlling for child, parent, and family characteristics. The findings suggest that parental health literacy could be a valuable element to consider in family-centered early childhood health promotion and preventive health education approaches.

## 1 Introduction

Since health-related knowledge, attitudes, and behaviors acquired in early childhood have a significant impact on later stages of life, it is crucial to explore the areas for the protection and development of health in the preschool period (1, 2). One of the most efficient ways to protect and enhance children’s health and instill healthy habits is through health education during preschool (3, 4). Health education constitutes a set of learning experiences crafted to enhance the knowledge and attitudes of individuals and communities, thereby promoting improvements in their health (5). According to the Centers for Disease Control and Prevention (CDC) Health Education Standards (6), health education should begin in kindergarten to foster health-promoting behaviors, and it is recommended that health education be mandatory in preschool settings (7). Health education from an early age can contribute to improving individual and public health and reducing health inequalities (8, 9).

Within the scope of health education, students acquire competencies in areas such as nutrition, mental health, hygiene, sleep, social relationships, self-care, neglect and abuse, personal safety, and first aid. Owing to its broad scope, health education is characterized by a multidimensional, interdisciplinary, dynamic, and functional structure (10, 11). Accordingly, it is widely recognized that health education during early childhood extends beyond a singular aspect of health; rather, it should be evaluated as a multidimensional, holistic structure encompassing physical, psychosocial, behavioral, and preventive health domains together. Indeed, the persistence of various health risks—such as obesity, diabetes, mental health problems, sleep disorders, excessive screen time, and sedentary lifestyle—during early childhood (12–19) further underscores the significance of this holistic approach. It is recommended that health education in early childhood be provided holistically through a multidimensional approach that integrates multiple disciplines, with active participation from children’s families (4). Children learn health-related knowledge, attitudes, behaviors, and skills through interactions with those in their immediate environment, such as family members, teachers, peers, the media, and health professionals.

The importance of parents as the primary influence in a child’s immediate environment can be explained by Bronfenbrenner’s microsystem theory of ecological systems and Bandura’s social cognitive theory of learning through observation and role modeling (20, 21). According to these theories, parents can be regarded as key actors and influential role models in the health-related learning environment of preschool children. Health behaviors—such as dietary habits, personal hygiene practices, sleep routines, the promotion of safe behaviors, and protection against various health-related risks—are shaped through daily interactions within the family environment during preschool years. The role of parents in children’s health education extends far beyond the transmission of information, encompassing opportunities for children to engage in healthy behaviors and support for maintaining these behaviors once learned. Because in the Predisposing, Reinforcing, and Enabling Constructs in Educational Diagnosis and Evaluation (PRECEDE) model, determinants of health education are classified as predisposing, enabling, and reinforcing factors (22). Based on this model, parents can play a predisposing role by supporting the development of health-related knowledge, attitudes, and values; an enabling role by providing the necessary skills, resources, environment, and opportunities for practice; and a reinforcing role by supporting healthy behaviors and providing feedback. Therefore, the transfer and maintenance of health-related knowledge and behaviors that children acquire in school or other social settings into their daily lives may depend on parents providing opportunities for practice and on their support of these behaviors.

The multifaceted nature of health education requires parents to possess the competencies needed to access accurate information across diverse health domains, critically evaluate it, and translate it into everyday practices. In this respect, health literacy can emerge as a crucial competency that may be linked to parents’ capacity to support their children’s health-related learning environment. This is because health literacy encompasses the competencies to access, comprehend, evaluate, and use information and services that promote and protect good health and wellbeing (23). A growing body of evidence has demonstrated significant relationships among health literacy, health education, and health behaviors, and that health literacy may serve as a mediating role in the formation of health behaviors (24–26). Furthermore, previous studies underline that the relationship between health literacy and health education is not unidirectional; health literacy can enhance health education processes and be shaped by health education outcomes (27, 28).

The literature contains findings demonstrating a link between parents’ general health literacy and children’s health and health-related behaviors. For instance, Aygun and Topcu (29) found that parental health literacy is associated with the health status and anthropometric indicators of children aged 0–6. Ogi et al. (30) also discovered a relationship between parental health literacy and children’s sleep duration. Moreover, Cheng et al. (31) found that low parental health literacy was independently associated with children’s television-viewing behavior and with family practices associated with injury risk. In addition, parental health literacy may be associated with health-promoting habits and parenting practices in early childhood (32, 33).

The literature addressing health education and parental health literacy together in the preschool period remains limited (4, 32, 34, 35). Although existing studies provide significant evidence, most focus on specific health areas or individual health outcomes, such as nutrition, food, oral health, sleep, vaccination, and fever management (30, 33). In contrast, the relationship between parental health literacy and multidimensional health education competencies across diverse domains among preschool-aged children has received insufficient attention in existing literature.

It has been noted that health literacy is correlated with socioeconomic characteristics such as education and income, and that the relationships with health outcomes persist even after these characteristics are taken into account (36–39). Therefore, it is of great significance to investigate the relationship between parental health literacy and children’s health education competencies while controlling for child-, parent-, and family-level characteristics and assessing the incremental explanatory contribution of health literacy to the model. Furthermore, considering that various domains within health education may be differentially associated with family routines, environmental conditions, and developmental processes, it is essential to analyze this relationship both from the perspective of overall health education competence and within specific subdomains. This study addresses two gaps in the literature by conceptualizing preschool children’s health education across six domains: personal safety and first aid, hygiene and self-care, nutrition, sleep, mental health and social relationships, and neglect and abuse, as well as examining the incremental explanatory contribution of parental health literacy beyond child-, parent-, and family-related characteristics. Accordingly, the following research questions were sought to answer by probing the characteristics of the child, parent, and family:

1) Is parental health literacy associated with parent-reported overall health education competency among preschool-aged children, and does it provide an incremental explanatory contribution to the model?
2) Is parental health literacy associated with each of the subdomains of health education?

## 2 Materials and methods

This study employed a cross-sectional design to examine the relationship between parental health literacy and health education scores among preschool children. It was conducted in preschools in Tokat, Türkiye, in 2024. The STROBE checklist for cross-sectional research was followed in reporting the study.

### 2.1 Population, sample, and participants

The study population consisted of 1,897 children aged 5–6 enrolled in approximately 110 kindergarten classes in the central district of Tokat Province, along with their parents. Considering the size of the target population, the minimum sample size was calculated to be approximately 320 using a maximum-variance assumption, a 95% confidence level, and a 5% margin of error. Accounting for potential data loss, the study aimed to recruit 400 participants.

The preschools in the research population were sorted using a random lottery method, and data were collected from these preschools in accordance with this order until the target sample size was reached. Accordingly, data collection forms were distributed to the parents of 600 children enrolled in 38 preschool classes. Only one parent was asked to participate in the study for each child, and the form was to be completed by the parent with the most knowledge of the child’s daily health, education, and care. This approach was adopted to prevent multiple parental reports for the same child and to obtain information from the parent who most closely monitors the child’s day-to-day care and health practices.

Inclusion criteria for the study were residing in the city center of Tokat, being the parent of a 5-6-year-old child attending preschool, and being literate at a level sufficient to comprehend and complete the data collection tools. In cases where a parent had more than one child aged 5–6 years, the parent was asked to provide responses for the oldest child, in months, to avoid obtaining multiple observations from the same parent.

The data were collected between April 8, 2024, and May 20, 2024, in cooperation with school administrators and preschool teachers, using a distribution-and-collection method (40). Of the 600 forms distributed to parents during data collection, 418 parents responded (response rate: 69.7%). After 11 forms with incomplete responses were excluded from the analysis, 407 parents comprised the final sample.

### 2.2 Data collection tools

The Health Literacy Scale-Short Form (HLS-SF12) was utilized to assess parental health literacy, and the parent-reported Health Education Scale for Preschool Children was used to evaluate children’s knowledge, skills, and behaviors related to health education. Furthermore, the Sociodemographic and Health Information Form, designed by the researchers, was used to identify child, parent, and family characteristics.

The HLS-SF12 is a 12-item measurement tool developed by Duong et al. (41) based on the European Health Literacy Scale (HLS-EU-Q47). Items are scored with four-category response options ranging from 1 (very difficult) to 4 (very easy). The health literacy index score is calculated using the formula “Index = (Mean − 1) × 50/3” and ranges from 0 to 50, with higher scores indicating greater health literacy. The HLS-SF12 assesses general adult health literacy and is not a tool specific to parenting or childcare. The Turkish validity and reliability study of the scale was carried out by Yılmaz and Eskici (42), and the Cronbach’s alpha coefficient was reported as 0.856. In this study, the Cronbach’s alpha coefficient of HLS-SF12 was found to be 0.88.

The Health Education Scale for Preschool Children was developed by Aydos (43) to evaluate the knowledge, skills, and behaviors of 5- to 6-year-old children in health education, using parental reports. The scale, which uses a 5-point Likert-type scale, has six subscales and a total of 117 items: personal safety and first aid (25 items), hygiene and self-care (26 items), nutrition (20 items), sleep (7 items), mental health and social relationships (20 items), and neglect and abuse (19 items). The overall scale score is calculated by dividing the sum of the item scores by 117, whereas the subscale scores are calculated by dividing the sum of the corresponding item scores by the number of items in the respective subscale. Overall and subscale scores range from 1 to 5, with higher scores indicating greater parent-reported knowledge, skills, and behaviors related to health education. In the development study of the scale, exploratory factor analysis was conducted using data from 456 parents, and confirmatory factor analysis was performed with data from 277 parents. The validity and reliability analyses of the subscales yielded Cronbach’s alpha coefficients ranging from 0.885 to 0.945 (43). In addition, psychometric findings for some subscales were reported in a subsequently published study (44). In the present research, Cronbach’s alpha coefficients for the overall scale and subscales ranged from 0.90 to 0.97.

The Sociodemographic and Health Information Form, developed by the researchers, contains 18 items designed to identify child-, parent-, and family-related characteristics. The form included questions about sociodemographic and health-related characteristics such as the child’s age, gender, chronic disease, and regular medication use, as well as the responding parent’s age, educational status, chronic disease, and smoking status, number of children, and perceived economic status. As reported by parents, it took nearly 45 min to complete the data-collection tools.

### 2.3 Research variable

The primary outcome variable of the study was the parent-reported overall health education score obtained using the Health Education Scale for Preschool Children. Scores obtained from the subscales-personal safety and first aid, hygiene and self-care, nutrition, sleep, mental health and social relationships, and neglect and abuse-were evaluated as domain-specific outcome variables. Overall scale and subscale scores were used as continuous variables in the analyses. The study’s main explanatory variable was parental health literacy, measured by the HLS-SF12 index score, which was treated as a continuous variable in the analyses. Child-related covariates were age, gender, chronic disease status, and regular medication use, while parent and family-related covariates were whether the respondent parent was the mother or father, parental age, education level, perceived economic status, number of children, parental chronic disease status, and smoking status. These covariates were incorporated into multivariate models, adjusted for child, parent, and family characteristics, to assess the relationships between parental health literacy and children’s parent-reported general and domain-specific health education scores.

### 2.4 Data analysis

The analyses were conducted on 407 participants who constituted the final analysis sample. Continuous variables were summarized using means, standard deviations, and minimum and maximum values, whereas categorical variables were summarized using frequencies and percentages. The median, skewness, and kurtosis values were calculated for the scale and its subscale scores.

A hierarchical multiple linear regression analysis was conducted to assess the adjusted association between parental health literacy and parent-reported overall health education scores among preschool-aged children, as well as the incremental explanatory contribution of parental health literacy beyond child-, parent-, and family-level characteristics. The variables were entered into the model using the Enter method in three theoretically determined blocks. Model 1 included the child’s age, gender, presence of chronic disease, and regular medication use, while Model 2 additionally consisted of whether the responding parent is the mother or father, the parent’s age, educational level, perceived economic status, number of children, the parent’s chronic disease conditions, and smoking status. In Model 3, the HLS-SF12 index score was added to assess the incremental explanatory contribution of parental health literacy beyond the variables included in the preceding blocks.

Given the final sample of 407 participants, a sensitivity analysis was conducted for the incremental *R*^2^ test using G*Power 3.1.9.7 (F tests; linear multiple regression: fixed model, *R*^2^ increase). With α = 0.05, 80% power, one tested predictor in the final block, and 16 total predictor degrees of freedom in the final model, the minimum detectable incremental effect size was Cohen’s *f*^2^ = 0.019.

The same three-block modeling approach was applied separately to each of the six domain-specific health education scores: personal safety and first aid; hygiene and self-care; nutrition; sleep; mental health and social relationships; and neglect and abuse. In order to control the false discovery rate caused by multiple tests, the Benjamini-Hochberg (BH) correction was applied to the *p*-values of the parental health literacy coefficient in these six models alone. The main model for the general health education score was not included in the BH adjustment.

Categorical variables were converted into dummy variables prior to regression analysis. The reference categories were defined as: male child; the responding parent being the mother; the absence of chronic disease in the child and the parent; the child not using regular medication; the parent not smoking; being a primary school graduate; and moderate perceived economic status. Child age was entered into the model as a numerical variable with values of 5 and 6 years, and the corresponding regression coefficient represents the mean score difference between 6-year-olds and 5-year-olds after adjustment for the other variables in the model. Parental age, number of children, and the HLS-SF12 index score were analyzed as continuous variables.

The explanatory power of the models was assessed using *R*^2^, the change in explained variance with the addition of blocks (Δ*R*^2^), and the corresponding *F*-test of variation (Δ*F*). Both the unstandardized regression coefficient (*B*) and its 95% confidence interval, as well as the standardized regression coefficient (*β*), were reported.

Prior to interpreting the regression models, model assumptions and potentially influential observations were assessed. Linearity and the assumption of constant residual variance (homoscedasticity) were assessed using scatterplots of standardized residuals against standardized predicted values, while the distribution of the residuals was examined using histograms and normal P–P plots. Autocorrelation in residuals was evaluated using the Durbin-Watson statistic, while multicollinearity was assessed using tolerance and variance inflation factor (VIF) values. Potentially influential observations were assessed using standardized residuals, Cook’s distance, and leverage values. The three observations were evaluated separately due to their high standardized residual values. They were found to contain neither data-entry nor scoring errors and, based on the joint assessment of influence diagnostics, were not considered excessively influential on the model. Therefore, all 407 participants were included in the analyses. In addition, Harman’s single-factor test was conducted using the combined 129-item pool of the HLS-SF12 and the Health Education Scale to provide a limited diagnostic of potential common method variance.

All statistical analyses were performed using IBM SPSS Statistics for Windows, Version 26.0. All statistical tests were two-tailed, and statistical significance was set at *p* < 0.05. Statistical inferences regarding parental health literacy in the six domain-specific models were based on BH-adjusted *p*-values.

### 2.5 Ethical approval and legal implementation permissions

Ethical approval was obtained from the Tokat Gaziosmanpaşa University Social and Human Sciences Research Ethics Committee before commencing the research (Approval No. 377723). To conduct the research in preschool education institutions, permission for research and implementation was obtained from the Provincial Directorate of National Education (Permission No. 95070948). Participation in the study was voluntary. Prior to data collection, parents were informed about the study’s purpose and procedures, and written consent was obtained from those who agreed to participate. In addition, the necessary permissions to use the HLS-SF12 and the Health Education Scale for Preschool Children in the study were obtained from the scale developers via email before data collection.

## 3 Results

Regarding the sociodemographic and health-related characteristics of participating parents, it was found that 80.3% of the study participants were mothers, and 19.7% were fathers. The mean age of the parents was 35.01 ± 4.97 years, with the highest education group being bachelor’s degree holders (31.9%). 90.7% of participants reported their perceived economic status as moderate. The rate of parents with chronic disease was 11.3%, and the proportion of smokers was 17.4%. The mean number of children per parent was 2.05 ± 0.79 (Table 1).

**Table 1:** Sociodemographic and health-related characteristics of participating parents (*n* = 407).

Characteristics (*n* = 407)	*n*	%
Responding parent		
Mother	327	80.3
Father	80	19.7
Educational status		
Primary education	66	16.2
High school	109	26.8
Associate degree	72	17.7
Bachelor’s degree	130	31.9
Master’s or doctoral degree	30	7.4
Perceived economic status		
Low	18	4.4
Moderate	369	90.7
Good	20	4.9
Chronic disease		
No	361	88.7
Yes	46	11.3
Smoking status		
No	336	82.6
Yes	71	17.4
Age (years) M ± SD, min-max	35.01 ± 4.97, 23–50	
Number of children M ± SD, min-max	2.05 ± 0.79, 1–5	

Mean: M, Standard Deviation: SD, minimum: min, Maximum: max. Column percentages are presented.

The characteristics of children are presented in Table 2. While 53.3% of the children were girls, 46.7% were boys. 54.8% of the children were 6 years old, and the mean age was 5.54 ± 0.49 years. It was reported that 6.4% of the children had a chronic disease and 4.9% took regular medication (Table 2).

**Table 2:** Child-related characteristics (*n* = 407).

Characteristics (*n* = 407)	*n*	%
Gender		
Female	217	53.3
Male	190	46.7
Age (years)		
5	184	45.2
6	223	54.8
Chronic disease		
No	381	93.6
Yes	26	6.4
Regular medication use		
No	387	95.1
Yes	20	4.9
Age (years) M ± SD, min-max	5.54 ± 0.49, 5–6	

Column percentages are presented.

Descriptive statistics regarding the participants’ HLS-SF12, overall health education, and subscale scores are shown in Table 3. The mean HLS-SF12 score was 38.41 ± 7.95, while the mean health education score was 4.14 ± 0.46. Among the subscales, the highest mean was in the neglect and abuse domain (4.26 ± 0.51), and the lowest in the nutrition domain (3.81 ± 0.67) (Table 3).

**Table 3:** Descriptive statistics and internal consistency of parental health literacy and parent-reported preschool health education scores.

Scale/subscale	Min	Max	Mean	SD	Median	Skewness	Kurtosis	*α*
HLS-SF12	12.50	50.00	38.41	7.95	37.50	−0.270	−0.407	0.88
Preschool Children’s Health Education Scale—overall score	2.53	5.00	4.14	0.46	4.13	−0.357	−0.019	0.97
Personal safety and first aid	2.20	5.00	4.21	0.53	4.28	−0.462	−0.393	0.94
Hygiene and self-care	2.04	5.00	4.21	0.51	4.19	−0.556	0.439	0.93
Nutrition	1.10	5.00	3.81	0.67	3.85	−0.602	0.379	0.92
Sleep	1.00	5.00	4.00	0.88	4.00	−0.893	0.609	0.90
Mental health and social relationships	2.45	5.00	4.22	0.51	4.20	−0.405	−0.129	0.93
Neglect and abuse	2.68	5.00	4.26	0.51	4.26	−0.369	−0.498	0.93

Health Literacy Scale-Short Form: HLS-SF12, Standard Deviation: SD, Cronbach’s alpha: *α*. Higher scores indicate higher levels of the respective constructs.

The results of the hierarchical multiple linear regression analysis examining whether parental health literacy was associated with parent-reported health education scale scores among preschool-aged children after controlling for child-, parent-, and family-related characteristics are presented in Table 4. In Model 1, child-related characteristics explained 32.5% of the variance in the health education scale score (*R*^2^ = 0.325; Δ*F* = 48.451; *p* < 0.001). In this model, only child age was found to be significant (*B* = 0.529; 95% CI [0.453–0.604]; *β* = 0.569; *p* < 0.001). On the other hand, the child’s gender, chronic disease status, and regular medication use were not significantly correlated with the health education scale score (Table 4).

**Table 4:** Hierarchical multiple linear regression models for the parent-reported Preschool Children’s Health Education Scale score.

Independent variables	Model 1: Child-related characteristics				Model 2: + Parent and family characteristics				Model 3: + Parental health literacy			
	*B*	*β*	95% CI for *B*	*p*	*B*	*β*	95% CI for *B*	*p*	*B*	*β*	95% CI for *B*	*p*
*(Constant)*	*1.226*	*_*	*[0.806, 1.645]*	<0.001	*1.592*	*-*	*[1.082, 2.103]*	<0.001	*1.271*	*-*	[*0.761, 1.780*]	<0.001
Child gender: female	−0.012	−0.013	[−0.088, 0.064]	0.755	−0.019	−0.021	[−0.096, 0.057]	0.617	−0.011	−0.012	[−0.085, 0.063]	0.769
Child age	**0.529**	**0.569**	**[0.453, 0.604]**	**<0.001**	**0.514**	**0.554**	**[0.437, 0.592]**	**<0.001**	**0.475**	**0.511**	**[0.398, 0.551]**	**<0.001**
Chronic disease in the child: yes	−0.077	−0.041	[−0.290, 0.137]	0.481	−0.100	−0.053	[−0.313, 0.113]	0.357	−0.058	−0.031	[−0.265, 0.149]	0.581
Regular medication use by the child: yes	−0.095	−0.044	[−0.333, 0.143]	0.433	−0.092	−0.043	[−0.331, 0.147]	0.448	−0.087	−0.041	[−0.318, 0.144]	0.461
Responding parent: father					−0.002	−0.001	[−0.109, 0.106]	0.977	−0.021	−0.018	[−0.125, 0.083]	0.690
Parent age					−0.002	−0.026	[−0.011, 0.006]	0.581	0.000	−0.004	[−0.009, 0.008]	0.924
Parent education: high school					−0.075	−0.072	[−0.195, 0.045]	0.221	−0.108	−0.104	[−0.225, 0.009]	0.069
Parent education: associate degree					−0.057	−0.047	[−0.188, 0.074]	0.394	−0.120	−0.099	[−0.250, 0.009]	0.068
Parent education: bachelor’s degree					−0.103	−0.104	[−0.227, 0.021]	0.103	**−0.143**	**−0.145**	**[−0.264, −0.022]**	**0.020**
Parent education: master’s or doctoral degree					**−0.277**	**−0.157**	**[−0.458, −0.096]**	**0.003**	**−0.310**	**−0.175**	**[−0.486, −0.134]**	**0.001**
Perceived economic status: low					−0.052	−0.023	[−0.236, 0.132]	0.581	−0.037	−0.016	[−0.215, 0.141]	0.683
Perceived economic status: good					0.120	0.056	[−0.055, 0.295]	0.178	0.148	0.069	[−0.022, 0.317]	0.088
Number of children					**−0.059**	**−0.101**	**[−0.113, −0.006]**	**0.030**	−0.050	−0.085	[−0.102, 0.002]	0.059
Chronic disease in the parent: yes					0.009	0.006	[−0.110, 0.128]	0.881	0.018	0.012	[−0.097, 0.134]	0.757
Parent smoking status: yes					0.027	0.022	[−0.076, 0.130]	0.607	0.032	0.026	[−0.068, 0.132]	0.534
Parental health literacy score									**0.012**	**0.215**	**[0.008, 0.017]**	**<0.001**
*R* ^2^	**0.325**				**0.353**				**0.395**			
*ΔR* ^2^	**0.325**				**0.028**				**0.041**			
*ΔF*(*p*)	**48.451, *p* < 0.001**				**1.546,** *p* = 0.113				**26.650, *p* < 0.001**			

The dependent variable was the parent-reported overall score on the Health Education Scale for Preschool Children. B, unstandardized regression coefficient; β, standardized regression coefficient; CI, confidence interval; *R*^2^, coefficient of determination; Δ*R*^2^, change in explained variance; ΔF, change in F statistic. Reference categories were male child, mother as the responding parent, absence of chronic disease in the child and parent, no regular medication use by the child, no parental smoking, primary education, and moderate perceived economic status. Durbin–Watson = 1.817. Bold regression estimates and their corresponding *p* values indicate statistically significant associations (*p* < 0.05). The model-summary values (*R*^2^, Δ*R*^2^, and ΔF) are also shown in bold; boldface in these rows does not by itself indicate statistical significance.

With the addition of parent- and family-related characteristics to Model 2, the explained variance increased from 32.5 to 35.3%. However, the 2.8% increase in explained variance was not statistically significant (Δ*R*^2^ = 0.028; Δ*F* = 1.546; *p* = 0.113). The coefficients for the variables in Model 2 are presented in Table 4.

In model 3, the inclusion of parental health literacy explained an additional 4.1% of the variance in the health education scale score (Δ*R*^2^ = 0.041; Δ*F* = 26.650; *p* < 0.001). After controlling for all child-, parent-, and family-related characteristics, parental health literacy was positively correlated with health education scale scores (*B* = 0.012; 95% CI [0.008–0.017]; *β* = 0.215; *p* < 0.001). Accordingly, each 1-point increase in the parental health literacy index was found to be associated with a 0.012-point increase in the child’s health education scale score.

Child age remained the variable with the strongest correlation with the health education scale score in the final model (*B* = 0.475; 95% CI [0.398–0.551]; *β* = 0.511; *p* < 0.001). When other variables were controlled for, the health education scale scores of 6-year-old children were found to be approximately 0.48 points higher than those of 5-year-old children. Compared with primary school graduate parents, parents with bachelor’s degrees (*B* = −0.143; 95% CI [−0.264 to −0.022]; *β* = −0.145; *p* = 0.020) and parents with master’s or doctoral degrees (*B* = −0.310; 95% CI [−0.486 to −0.134]; *β* = −0.175; *p* = 0.001) reported lower health education scale scores for their children (Table 4). In the final model for the overall health education score, VIF values ranged from 1.04 to 2.48 and tolerance values ranged from 0.40 to 0.96; for parental health literacy, the VIF was 1.11 and the tolerance value was 0.90.

In the final models of the hierarchical regression analyses for the six subscales, child age was positively associated with higher scores across all subscales and had the largest absolute standardized regression coefficient in each model (*B* = 0.395–0.529; *β* = 0.223–0.475; all *p* < 0.001). Detailed results for other covariates and all model stages are presented in Supplementary Tables S1–S6.

In the subscale analyses, higher parental health literacy was significantly associated with higher scores on the personal safety and first aid (*B* = 0.014; 95% CI [0.008–0.020]; *β* = 0.208; BH-adjusted *p* < 0.001), hygiene and self-care (*B* = 0.011; 95% CI [0.005–0.016]; *β* = 0.165; BH-adjusted *p* < 0.001), nutrition (*B* = 0.012; 95% CI [0.004–0.020]; *β* = 0.145; BH-adjusted *p* = 0.003), mental health and social relationships (*B* = 0.014; 95% CI [0.008–0.019]; *β* = 0.207; BH-adjusted *p* < 0.001), and neglect and abuse (*B* = 0.015; 95% CI [0.010–0.021]; *β* = 0.234; BH-adjusted *p* < 0.001) subscales after Benjamini–Hochberg adjustment. In the sleep subscale, no significant relationship was found between parental health literacy and scores (*B* = 0.004; 95% CI [−0.007 to 0.015]; *β* = 0.036; BH-corrected *p* = 0.481). The identified themes are presented in Figure 1.

**Figure 1:**
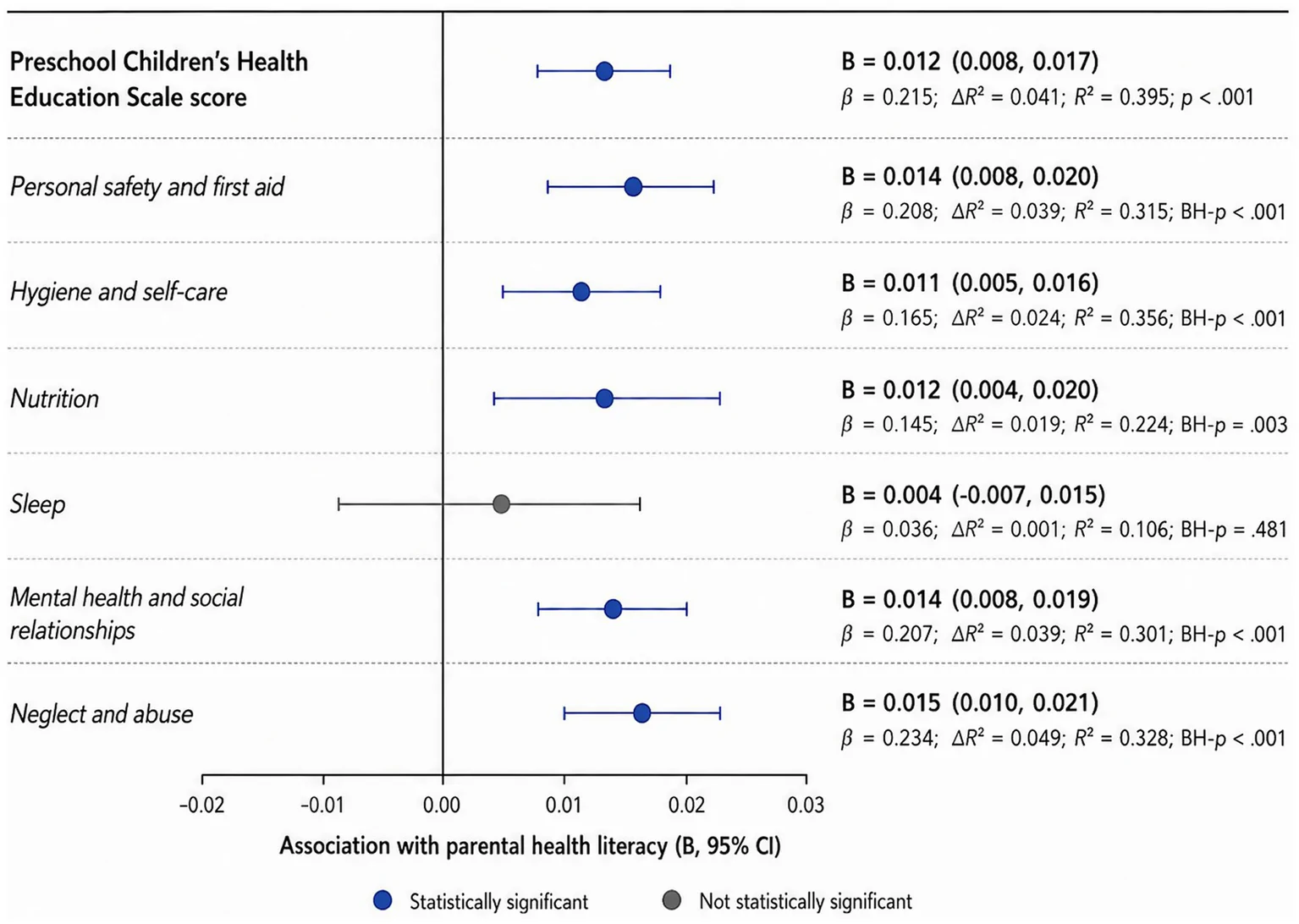
Multivariable-adjusted associations of parental health literacy with the parent-reported overall and subscale scores of the Health Education Scale for Preschool Children (Points and horizontal lines represent unstandardized regression coefficients (*B*) and 95% confidence intervals. All estimates are from the final models adjusted for child-, parent-, and family-related characteristics. *β* represents the standardized regression coefficient. Δ*R*^2^ represents the additional variance explained by parental health literacy after its addition to the model, and *R*^2^ represents the variance explained by the final model. Benjamini–Hochberg-adjusted *p* values are reported only for the six subscale analyses; the *p* value for the overall score is unadjusted).

## 4 Discussion

The fact that parental health literacy provides an additional 4.1% explanatory power to the variance in the general health education score in a model that accounts for child, parent, and family-related characteristics indicates that the relationship between parental health literacy and preschool children’s health education competencies is not limited to a single health domain but exhibits a broader pattern of association. More importantly, similar relationships persisted in five of six health education domains after the Benjamini-Hochberg correction, indicating that this relationship pattern is not limited to specific areas such as nutrition or hygiene. Conversely, the lack of a significant association in the sleep domain suggests that the relationship with parental health literacy may not occur similarly across all areas of health education. When the research results are evaluated as a whole, it can be noted that parental health literacy is a feature that should be considered within the multifaceted structure of preschool health education, but it does not constitute an adequate explanation on its own.

Previous studies examining the links between parental health literacy and children’s health behaviors are generally consistent with these findings, with higher parental health literacy associated with more positive health behaviors in preschool children (32). In their systematic review, Csima et al. (33) revealed that research on parental health literacy and the health of children under five focuses on individual health domains (food, nutrition, oral health, vaccination, and fever management) rather than overall health outcomes, and that parental health literacy is associated with the management of childhood diseases. Li et al. (45), on the other hand, concluded in their systematic review that maternal health literacy is a potential determinant of early childhood health and that existing studies have explored various areas of health. Other systematic reviews (2, 46) noted that low parental health literacy is correlated with poor child health outcomes. Previous research has also examined the association between parental health literacy and health education competencies in early childhood, with many focusing on individual health domains, such as sleep disorders (47), nutrition (48), body mass index (49, 50), child abuse (51), and injury (31). The present study extends the existing literature by evaluating diverse domains of health education—including personal safety and first aid, mental health, nutrition, hygiene and self-care, sleep, and neglect and abuse—within a common analytical framework. Thus, it facilitated the assessment of whether the relationship framework linked to parental health literacy extends to a comprehensive preschool health education profile, surpassing solely individual health outcomes.

The fact that the relationship between parental health literacy and health education scores persists after adjusting for multiple comparisons across five different domains—personal safety and first aid, hygiene and self-care, nutrition, mental health and social relationships, and protection from neglect and abuse—implies that the relationship structure associated with parental health literacy extends to health education competencies of varying nature. Although domains of health education vary in content and the required skills, the fact that they encompass not only accessing and comprehending health information but also evaluating and applying it in everyday life provides a shared conceptual framework for understanding the interrelations among these fields. In this respect, although these domains fall under the umbrella term of health education, each comprises distinct processes, underscoring the need for domain-specific assessment.

The finding regarding the relationship between personal safety and first aid corroborates with similar studies in the literature. Parental education is a key factor in ensuring child safety and preventing accidents and injuries (52), and parents’ level of health literacy affects children’s safety (53). The literature provides evidence that parents with higher levels of health literacy are more likely to integrate accident prevention and safety-related activities into their children’s daily routines (32); lower parental health literacy is associated with a higher risk of child injury (31); maternal health literacy predicts children’s knowledge of traffic accident prevention (54); first-aid knowledge increases with parental literacy (55); and parental health literacy related to unintentional injuries among young children is generally low (56). The relationship observed within the domain of personal safety and first aid in this study indicates that it may be associated with parents’ assessment of health and safety-related information, their recognition of risks, and the implementation of this information into daily preventive practices.

Further, the findings in the domain of hygiene and self-care highlight the significance of parental health literacy for children’s daily health routines. Although the literature contains limited research directly comparable to the present study in terms of parental health literacy, existing studies have predominantly focused on specific domains, such as oral health. The family constitutes children’s primary environment where self-care skills are reinforced and acquired (57). The roles parents play as role models in the family setting—such as their health knowledge and toothbrushing habits—influence children’s health-related knowledge and behaviors (58). It has been reported that children of parents with high levels of health literacy exhibit more positive health behaviors, such as regular toothbrushing (59, 60). These previous studies did not assess the same construct as the hygiene and self-care competencies examined in the present study; nonetheless, they acknowledge the potential importance of parental efforts in converting health-related information into daily routines and in guiding their children through these processes.

Additionally, parents with high levels of health literacy are more likely to guide their children toward healthy diets (61), and their children demonstrate more positive health behaviors associated with healthy eating (48, 59). These findings imply that the correlation between parental health literacy and the nutrition domain identified in this study may pertain to parents’ capacity to assess health and nutrition information, integrate this knowledge into their daily food selections and routines, and steer their children toward adopting health-promoting behaviors. Moreover, the nutrition domain may represent one of the most visible contexts in which parental health literacy is translated into children’s everyday health-learning environment, potentially because eating is an activity that parents and children frequently engage in together.

Parents play a vital role in supporting their children’s mental health and managing their help-seeking behaviors (62). Despite limited research on parental health literacy and preschool children’s mental health competencies, parent–child interaction emerges as a significant factor in studies of early childhood mental health literacy (63). The findings in this study concerning the relationship between parental health literacy and mental health and social relationships may be linked to parental competencies and practices, since competencies such as establishing family social interaction, managing coping strategies, seeking help, and emotion regulation, which include dynamic and reciprocal skills, can be shaped in children with parental practices. For this reason, it is believed that parental health literacy—in terms of children’s health—may extend beyond areas such as nutrition and self-care to include processes such as recognizing emotional signs, interpreting behaviors, defining appropriate support, and communicating with the child about health-related issues.

With regard to the domain of protection from neglect and abuse, it is deemed that the observed relationship can be linked to the role of parental health literacy in protecting children’s health. Relatively few studies have investigated parental health literacy and children’s knowledge of neglect and abuse. Zhang et al. (64) uncovered that parental communication with their children is an important factor in preventing preschool child abuse. Further, Apaydın Cırık et al. (65) found that children of parents who provided information on sexual abuse prevention had better knowledge of what to do in the event of abuse. In this regard, parents are key determinants of how their children learn about body safety, personal boundaries, private areas, safe touch, and help-seeking. The relationship between parental health literacy and the domain of protection from neglect and abuse indicates that health literacy may also be related to the context of safety and protection from a broader perspective.

Importantly, the relatively small incremental explanatory contribution of parental health literacy to the sleep domain, together with the lack of a statistically significant association after adjustment for multiple comparisons, reveals that the relationship between health literacy and children’s health education does not consistently manifest across all domains. This result is inconsistent with some studies that have investigated the relationship between parental health literacy and child sleep. Indeed, previous research has largely centered on the relationships between parental health literacy and children’s sleep duration and behaviors. In their study, Ogi et al. (30) found a positive correlation between preschool children’s sleep duration and parents’ low health literacy; however, no association was observed in the group with high parental health literacy. This finding indicates that the relationship between parental health literacy and child sleep is linear and does not occur in the same way at all health literacy levels. According to Ono et al. (47), parents’ health literacy predicts preschool children’s sleep problems. Khalili et al. (66) also reported that higher levels of maternal health literacy were associated with better sleep habits in children. Bonuck et al. (67) provided sleep health education to preschool children and their parents; although a slight increase in parental knowledge was observed following the intervention, no significant effect on the children’s sleep behaviors was found.

Taken together, the link between parental health literacy and children’s sleep knowledge has not been directly tested in most studies, and evidence regarding parental health literacy and children’s sleep problems, sleep duration, or sleep hygiene practices varies, limiting the consistency of results across studies.

Unlike other domains of health education, sleep is influenced by numerous factors—including a child’s developmental characteristics, established sleep patterns, bedtime routines, screen time, the sleep environment, and the family’s daily schedule—in addition to how parents apply their health knowledge in daily life. These factors may impact children’s sleep behaviors either independently of or in conjunction with parental health literacy. Moreover, given that children’s and parents’ sleep needs and patterns may not coincide, the influence of modeling and shared daily routines—as observed in other health behaviors—may be more limited for sleep. In addition, 87 of 407 participants (21.4%) attained the maximum sleep-subscale score, indicating some degree of upper-end clustering; however, this finding alone is insufficient to conclude that the null association resulted from a ceiling effect. At the same time, the absence of a substantial correlation between parental health literacy and the sleep domain indicates that an evaluation should be undertaken, taking into account individual, familial, and environmental factors pertinent to sleep.

Of particular note, one variable that demonstrates a strong correlation with health education competencies is child age. The fact that the highest standardized regression coefficient in the final model is for the child’s age, and that age was associated with higher scores across all health education domains, illustrates that preschool health education competencies cannot be assessed independently of the developmental context. The higher overall health education scores observed among 6-year-old children compared with those of 5-year-olds suggest that health-related knowledge, skills, and behaviors in this age range may evolve in tandem with developmental changes. In the study in which the Health Education Scale for Preschool Children was developed, it was observed that the general health education scores of 6-year-old children were higher than those of 5-year-old children (43). Furthermore, previous research revealed that health literacy increases with age among primary school children (68).

Another notable finding in this study was that parents with bachelor’s and master’s/doctoral degrees reported lower general health education scores for their children compared to parents with only a primary school education. This unanticipated finding illustrates the context-dependent differences that occur after controlling for coefficients, parental health literacy, and other child-, parent-, and family-related characteristics. Notably, the zero-order correlations of parental health literacy with the bachelor’s degree and master’s/doctoral degree indicators were negligible (r = 0.016 and r = −0.023, respectively), providing no evidence for a classical suppression interpretation. As a matter of fact, evidence links higher parental education and health literacy to more positive health habits during the preschool period (32). Similarly, in the original development study of the Preschool Children’s Health Education Scale, the overall scores did not differ by the educational level of the mother or father (43). Furthermore, in a recent parent-proxy study, higher parental education was found to be associated with a lower parent-reported child quality-of-life score in the adjusted model (69). Taken together, these findings suggest that the observed inverse associations should be interpreted as conditional relationships that may reflect sample characteristics, measurement factors, and model specification rather than as evidence of a general educational effect. On the other hand, it has been shown that there may be incomplete harmony between parent-proxy assessments and children’s own reports, particularly in domains that are less directly observable (70, 71). Recently, Wiedermann et al. (72) have stated that parental health literacy is associated with various parental-proxy child outcomes regardless of educational status and other sociodemographic characteristics, and parental characteristics may affect how the child’s condition is perceived and reported. For this reason, inverse education coefficients should be tested using studies that collect data from the child firsthand.

In the present study, domain-specific relationships were also detected for some covariates. An increase in the number of children was uncovered to be associated with lower scores in the domains of personal safety, first aid, and protection from neglect and abuse. Previous findings on the family context also suggest that the number of siblings alone does not provide an adequate explanation. It has been noted that the risk of injury among school-aged children can vary with sibling characteristics (73). What is more, the number of children did not display a significant relationship in the final overall health education model. The fact that parental smoking was associated with higher scores in the domain of protection from neglect and abuse is an incidental finding and should not be interpreted as a protective relationship. Furthermore, because the multiple-comparison adjustment was applied only to the parental health literacy coefficients in the subscale models, the domain-specific associations observed for the number of children and smoking should be regarded as secondary and exploratory findings that require confirmation in different samples.

### 4.1 Implications for public health and health education in preschool children

A significant implication of this study for public health is that parental health literacy must be considered when developing health education strategies for preschool children. Systematic reviews analyzing health literacy interventions for parents reveal that both in-person approaches and digital applications possess the potential to enhance parental health literacy, thereby augmenting parents’ health knowledge and influencing certain health-related behaviors (74, 75). The relationships observed in this study suggest that promoting parental health literacy could be a meaningful area of intervention in preschool health education.

The ongoing relationship between parental health literacy across various health education domains, even after accounting for multiple comparisons, suggests that family-centered strategies may be advantageous for addressing the diverse health requirements in preschool settings. In this sense, interventions aimed at supporting parental health literacy can be considered an important family-centered approach that can indirectly contribute to children’s health education competencies. It is recommended to test the extent to which parental health literacy is reflected in child-level outcomes using controlled, longitudinal studies.

### 4.2 Strengths and limitations

One of the strengths of this research is that it evaluated domains encompassing personal safety, first aid, hygiene, self-care, nutrition, sleep, mental health, social relationships, and protection from neglect and abuse in preschool children’s health education, all within the same analytical framework. This multidimensional approach facilitated the independent evaluation of the relationship between parental health literacy and each specific health domain, in addition to the overall health education score. Following the hierarchical integration of child-, parent-, and family-related characteristics into the model, the inclusion of parental health literacy facilitated the evaluation of this variable’s incremental explanatory contribution beyond these attributes. Beyond this, the separate construction of general and domain-specific models, the reporting of coefficients along with confidence intervals, the systematic evaluation of regression assumptions and influential observations, and the application of Benjamini-Hochberg correction to parental health literacy coefficients for six domains can be highlighted as other strengths of the analytical approach. The high internal consistency of the scales in the current sample also supported the reliability of the measurements. Furthermore, during field implementation, the preschool classes within the study population were randomly ordered via a lottery procedure, and this predetermined order was followed until the target sample size was reached, thereby limiting the researchers’ discretion in selecting classes for inclusion in the study.

With regard to the limitations, the cross-sectional design of the study did not allow for determining the temporal nature of the relationship between parental health literacy and children’s parent-reported health education scores, nor did it permit causal inferences. The possibility that the relationship is inverse or bidirectional cannot be ruled out, nor can the potential influence of unmeasured shared familial or environmental characteristics on both constructs. In addition, the scores for children’s health education reflected parent-reported evaluations. Obtaining parental health literacy and children’s health education scores from the same respondent may have led to the possibility of common method bias or social desirability effects (76). Nevertheless, Harman’s single-factor test showed that the first unrotated factor accounted for 28.2% of the total variance across the 129 items; however, this test provides only a limited diagnostic and cannot rule out common method bias entirely. Additionally, given the substantial number of items included in the combined data-collection instruments, some degree of response fatigue cannot be completely excluded, although no direct evidence of systematically declining response quality across later questionnaire sections was observed.

The fact that the research was conducted in preschools in a single city limits the generalizability of the findings to different geographical, rural, cultural, and socioeconomic contexts. Given the approximately 70% response rate, non-responding parents may have differed systematically from participating parents. The fact that the majority of respondents reported their perceived economic status as moderate, and that the economic situation was assessed using a subjective, categorical measure, may also have limited the detailed examination of socioeconomic differences and the accuracy of estimates for extreme categories. Because clustering of participants across preschool classes was not accounted for in the regression models, it may have affected the standard errors.

Although the Preschool Children’s Health Education Scale provides a multidimensional assessment, the health education domains examined in the present study were limited to those represented in the instrument and therefore do not encompass all potentially relevant areas of early childhood health education. Moreover, the HLS-SF12 assessed parents’ general health literacy (41) but did not measure health literacy specific to childcare or preschool child health. Thus, the findings should be interpreted in the context of parents’ capacity to access, understand, evaluate, and use general health information, rather than in the context of health literacy specific to children’s health. The effect of unmeasured confounders cannot be discounted since some variables that may be related to both parental health literacy and children’s health education scores, such as the child’s preschool attendance time, school and teacher characteristics, health-related practices within the family, and parents’ own health behaviors, were not evaluated in the study. Since multiple-comparison correction was applied only to parental health literacy coefficients in the subscale analyses, domain-specific relationships observed in other covariates should be considered secondary and exploratory findings.

Future research could utilize multicenter and longitudinal designs to assess the consistency and temporal aspect of observed relationships across different contexts. Assessing children’s health-related knowledge, skills, and behaviors using age-appropriate assessment tools—directly and in educational or real-life settings that are as natural as possible—can help isolate potential biases associated with parental reporting. The use of health literacy measures specific to parenting or child health, together with the application of multilevel models that account for clustering, may enable a more detailed examination of the relative contributions of child-, family-, and educational-environment factors.

## 5 Conclusion

The fact that parental health literacy offers additional explanatory contribution to the overall health education score beyond the measured characteristics of the child, parent, and family, and that this relationship is observed in domains such as personal safety, first aid, hygiene, self-care, nutrition, mental health, social relationships, and protection from neglect and abuse—but not in the domain of sleep—indicates that this relationship may vary depending on specific health domains. In this regard, it is evident that the relationship between parental health literacy and health education for preschool children is not limited to a single area of health but extends to various areas within the framework of multidimensional health education. The limited scope of multidimensional research on health in early childhood necessitates further studies that examine multiple areas of health education together, as well as the development of age-appropriate measurement tools to evaluate these areas. In future studies, supplementing parent-reported measures with direct assessments of children’s health-related knowledge, skills, and behaviors, preferably in their naturalistic settings, may contribute to a more valid understanding of relationships across domains and developmental differences. Since the current study design—based on cross-sectional and parental reports—does not allow for causal inferences regarding the direction of these relationships, the findings must be tested in multicenter and longitudinal studies.

## Data Availability

The de-identified data supporting the findings of this study are available from the corresponding author, subject to applicable ethical and institutional requirements.
