# Supplementary material for: Parental health literacy and multidimensional health education competencies in preschool children
Source: Front Public Health. 2026 Oct 29;14:1984037. doi: 10.3389/fpubh.2026.1984037 (PMC13755309; doi:10.3389/fpubh.2026.1984037)
Supplement: Supplementary file 1 [file Table_1.DOCX]

Supplementary Material

# 1 Supplementary Tables

**Supplementary Table S1.** Hierarchical multiple linear regression models for the personal safety and first aid subscale score

|  | **Model 1: Child-related characteristics** | | | | **Model 2: + Parent and family characteristics** | | | | **Model 3: + Parental health literacy** | | | |
| --- | --- | --- | --- | --- | --- | --- | --- | --- | --- | --- | --- | --- |
| **Independent variables** | **B** | **β** | **95% CI for B** | **p** | **B** | **β** | **95% CI for B** | **p** | **B** | **β** | **95% CI for B** | **p** |
| *(Constant)* | 1.359 | - | [0.839, 1.879] | <0.001 | 1.630 | - | [1.002, 2.257] | <0.001 | 1.268 | - | [0.639, 1.898] | <0.001 |
| Child sex: female | 0.024 | 0.022 | [-0.070, 0.118] | 0.616 | 0.013 | 0.012 | [-0.081, 0.107] | 0.789 | 0.022 | 0.021 | [-0.069, 0.114] | 0.634 |
| Child age | **0.514** | **0.476** | **[0.420, 0.607]** | **<0.001** | **0.505** | **0.468** | **[0.410, 0.601]** | **<0.001** | **0.461** | **0.427** | **[0.366, 0.556]** | **<0.001** |
| Chronic disease in the child: yes | -0.004 | -0.002 | [-0.268, 0.260] | 0.977 | -0.023 | -0.011 | [-0.285, 0.239] | 0.861 | 0.023 | 0.011 | [-0.233, 0.279] | 0.857 |
| Regular medication use by the child: yes | -0.173 | -0.070 | [-0.468, 0.121] | 0.248 | -0.194 | -0.078 | [-0.487, 0.100] | 0.195 | -0.188 | -0.076 | [-0.474, 0.098] | 0.197 |
| Responding parent: father |  |  |  |  | -0.023 | -0.017 | [-0.155, 0.108] | 0.727 | -0.045 | -0.034 | [-0.174, 0.083] | 0.488 |
| Parent age |  |  |  |  | 0.003 | 0.028 | [-0.008, 0.014] | 0.578 | 0.005 | 0.049 | [-0.005, 0.016] | 0.319 |
| Parent education: high school |  |  |  |  | 0.003 | 0.003 | [-0.144, 0.151] | 0.966 | -0.035 | -0.029 | [-0.179, 0.110] | 0.638 |
| Parent education: associate degree |  |  |  |  | -0.016 | -0.012 | [-0.177, 0.145] | 0.843 | -0.088 | -0.062 | [-0.247, 0.072] | 0.281 |
| Parent education: bachelor's degree |  |  |  |  | -0.104 | -0.091 | [-0.257, 0.048] | 0.179 | -0.150 | -0.130 | [-0.299, 0.000] | 0.050 |
| Parent education: master's or doctoral degree |  |  |  |  | **-0.255** | **-0.124** | **[-0.477, -0.032]** | **0.025** | **-0.292** | **-0.142** | **[-0.509, -0.074]** | **0.009** |
| Perceived economic status: low |  |  |  |  | -0.121 | -0.046 | [-0.347, 0.105] | 0.292 | -0.105 | -0.040 | [-0.325, 0.115] | 0.350 |
| Perceived economic status: good |  |  |  |  | 0.080 | 0.032 | [-0.135, 0.295] | 0.465 | 0.111 | 0.045 | [-0.098, 0.321] | 0.298 |
| Number of children |  |  |  |  | **-0.116** | **-0.170** | **[-0.181, -0.050]** | **0.001** | **-0.105** | **-0.155** | **[-0.169, -0.041]** | **0.001** |
| Chronic disease in the parent: yes |  |  |  |  | -0.011 | -0.006 | [-0.157, 0.135] | 0.884 | -0.001 | 0.000 | [-0.143, 0.142] | 0.994 |
| Parent smoking status: yes |  |  |  |  | -0.124 | -0.088 | [-0.251, 0.003] | 0.056 | -0.119 | -0.084 | [-0.243, 0.005] | 0.060 |
| Parental health literacy score |  |  |  |  |  |  |  |  | **0.014** | **0.208** | **[0.008, 0.020]** | **<0.001** |
| ***R²*** | **0.233** | | | | **0.276** | | | | **0.315** | | | |
| ***ΔR²*** | **0.233** | | | | **0.043** | | | | **0.039** | | | |
| **ΔF, (p)** | **30.532, p<.001** | | | | **2.133, p=0.017** | | | | **22.023, p<.001** | | | |
| Dependent variable: personal safety and first aid subscale score; B, unstandardized regression coefficient; β, standardized regression coefficient; CI, confidence interval; R², coefficient of determination; ΔR², change in explained variance; ΔF, change in F statistic. Reference categories were male child, mother, absence of chronic disease, no regular medication use, no smoking, primary education, and moderate economic status. Durbin-Watson: 1.930. Bold values indicate p<.05. | | | | | | | | | | | | |

**Supplementary Table S2.** Hierarchical multiple linear regression models for the hygiene and self-care subscale score

|  | **Model 1: Child-related characteristics** | | | | **Model 2: + Parent and family characteristics** | | | | **Model 3: + Parental health literacy** | | | |
| --- | --- | --- | --- | --- | --- | --- | --- | --- | --- | --- | --- | --- |
| **Independent variables** | **B** | **β** | **95% CI for B** | **p** | **B** | **β** | **95% CI for B** | **p** | **B** | **β** | **95% CI for B** | **p** |
| *(Constant)* | 1.113 | - | [0.628, 1.597] | <0.001 | 1.572 | - | [0.989, 2.155] | <0.001 | 1.296 | - | [0.706, 1.886] | <0.001 |
| Child sex: female | 0.037 | 0.036 | [-0.050, 0.124] | 0.404 | 0.039 | 0.037 | [-0.048, 0.126] | 0.380 | 0.046 | 0.044 | [-0.040, 0.132] | 0.291 |
| Child age | **0.557** | **0.534** | **[0.469, 0.644]** | **<0.001** | **0.529** | **0.508** | **[0.441, 0.618]** | **<0.001** | **0.495** | **0.475** | **[0.407, 0.584]** | **<0.001** |
| Chronic disease in the child: yes | -0.037 | -0.018 | [-0.284, 0.209] | 0.765 | -0.074 | -0.035 | [-0.317, 0.170] | 0.551 | -0.038 | -0.018 | [-0.278, 0.202] | 0.756 |
| Regular medication use by the child: yes | -0.031 | -0.013 | [-0.305, 0.244] | 0.826 | -0.031 | -0.013 | [-0.304, 0.241] | 0.822 | -0.027 | -0.011 | [-0.295, 0.241] | 0.845 |
| Responding parent: father |  |  |  |  | -0.037 | -0.028 | [-0.159, 0.085] | 0.552 | -0.054 | -0.041 | [-0.174, 0.067] | 0.380 |
| Parent age |  |  |  |  | -0.002 | -0.017 | [-0.012, 0.008] | 0.727 | -0.000 | 0.000 | [-0.010, 0.010] | 0.997 |
| Parent education: high school |  |  |  |  | -0.068 | -0.058 | [-0.205, 0.068] | 0.327 | -0.097 | -0.083 | [-0.233, 0.038] | 0.158 |
| Parent education: associate degree |  |  |  |  | -0.064 | -0.047 | [-0.214, 0.085] | 0.398 | -0.119 | -0.088 | [-0.269, 0.031] | 0.118 |
| Parent education: bachelor's degree |  |  |  |  | **-0.168** | **-0.151** | **[-0.309, -0.027]** | **0.020** | **-0.203** | **-0.182** | **[-0.343, -0.063]** | **0.005** |
| Parent education: master's or doctoral degree |  |  |  |  | **-0.404** | **-0.203** | **[-0.611, -0.197]** | **<0.001** | **-0.432** | **-0.218** | **[-0.636, -0.228]** | **<0.001** |
| Perceived economic status: low |  |  |  |  | 0.018 | 0.007 | [-0.192, 0.228] | 0.866 | 0.031 | 0.012 | [-0.176, 0.237] | 0.771 |
| Perceived economic status: good |  |  |  |  | 0.077 | 0.032 | [-0.122, 0.276] | 0.449 | 0.101 | 0.042 | [-0.096, 0.297] | 0.314 |
| Number of children |  |  |  |  | **-0.065** | **-0.099** | **[-0.126, -0.004]** | **0.037** | -0.057 | -0.086 | [-0.117, 0.003] | 0.063 |
| Chronic disease in the parent: yes |  |  |  |  | 0.013 | 0.008 | [-0.123, 0.148] | 0.856 | 0.020 | 0.012 | [-0.113, 0.154] | 0.764 |
| Parent smoking status: yes |  |  |  |  | 0.011 | 0.008 | [-0.107, 0.129] | 0.857 | 0.015 | 0.011 | [-0.101, 0.131] | 0.802 |
| Parental health literacy score |  |  |  |  |  |  |  |  | **0.011** | **0.165** | **[0.005, 0.016]** | **<0.001** |
| ***R²*** | **0.287** | | | | **0.331** | | | | **0.356** | | | |
| ***ΔR²*** | **0.287** | | | | **0.044** | | | | **0.024** | | | |
| **ΔF, (p)** | **40.513, p<.001** | | | | **2.345, p=0.008** | | | | **14.704, p<.001** | | | |
| Dependent variable: hygiene and self-care subscale score; B, unstandardized regression coefficient; β, standardized regression coefficient; CI, confidence interval; R², coefficient of determination; ΔR², change in explained variance; ΔF, change in F statistic. Reference categories were male child, mother, absence of chronic disease, no regular medication use, no smoking, primary education, and moderate economic status. Durbin-Watson: 1.847. Bold values indicate p<.05. | | | | | | | | | | | | |

**Supplementary Table S3.** Hierarchical multiple linear regression models for the nutrition subscale score

|  | **Model 1: Child-related characteristics** | | | | **Model 2: + Parent and family characteristics** | | | | **Model 3: + Parental health literacy** | | | |
| --- | --- | --- | --- | --- | --- | --- | --- | --- | --- | --- | --- | --- |
| **Independent variables** | **B** | **β** | **95% CI for B** | **p** | **B** | **β** | **95% CI for B** | **p** | **B** | **β** | **95% CI for B** | **p** |
| *(Constant)* | 0.611 | - | [-0.060, 1.282] | 0.074 | 0.974 | - | [0.150, 1.799] | 0.021 | 0.659 | - | [-0.181, 1.499] | 0.124 |
| Child sex: female | -0.077 | -0.057 | [-0.198, 0.044] | 0.212 | -0.098 | -0.072 | [-0.221, 0.026] | 0.120 | -0.089 | -0.066 | [-0.212, 0.033] | 0.151 |
| Child age | **0.587** | **0.434** | **[0.466, 0.708]** | **<0.001** | **0.568** | **0.420** | **[0.443, 0.693]** | **<0.001** | **0.529** | **0.391** | **[0.403, 0.655]** | **<0.001** |
| Chronic disease in the child: yes | -0.134 | -0.049 | [-0.475, 0.207] | 0.439 | -0.150 | -0.054 | [-0.494, 0.195] | 0.393 | -0.109 | -0.040 | [-0.450, 0.233] | 0.531 |
| Regular medication use by the child: yes | -0.018 | -0.006 | [-0.399, 0.362] | 0.925 | -0.004 | -0.001 | [-0.390, 0.382] | 0.984 | 0.001 | 0.000 | [-0.380, 0.383] | 0.995 |
| Responding parent: father |  |  |  |  | 0.029 | 0.017 | [-0.144, 0.202] | 0.746 | 0.009 | 0.005 | [-0.162, 0.181] | 0.915 |
| Parent age |  |  |  |  | -0.003 | -0.019 | [-0.016, 0.011] | 0.716 | -0.001 | -0.004 | [-0.014, 0.013] | 0.932 |
| Parent education: high school |  |  |  |  | -0.182 | -0.120 | [-0.375, 0.012] | 0.066 | **-0.215** | **-0.141** | **[-0.407, -0.022]** | **0.029** |
| Parent education: associate degree |  |  |  |  | -0.075 | -0.043 | [-0.287, 0.136] | 0.485 | -0.138 | -0.078 | [-0.351, 0.076] | 0.205 |
| Parent education: bachelor's degree |  |  |  |  | -0.105 | -0.073 | [-0.305, 0.095] | 0.301 | -0.145 | -0.100 | [-0.344, 0.055] | 0.154 |
| Parent education: master's or doctoral degree |  |  |  |  | -0.277 | -0.107 | [-0.569, 0.016] | 0.064 | **-0.309** | **-0.120** | **[-0.599, -0.019]** | **0.037** |
| Perceived economic status: low |  |  |  |  | -0.071 | -0.022 | [-0.368, 0.226] | 0.638 | -0.057 | -0.017 | [-0.351, 0.237] | 0.704 |
| Perceived economic status: good |  |  |  |  | 0.245 | 0.079 | [-0.037, 0.527] | 0.089 | 0.272 | 0.087 | [-0.008, 0.552] | 0.056 |
| Number of children |  |  |  |  | -0.028 | -0.033 | [-0.114, 0.058] | 0.517 | -0.019 | -0.022 | [-0.105, 0.066] | 0.659 |
| Chronic disease in the parent: yes |  |  |  |  | -0.022 | -0.011 | [-0.215, 0.170] | 0.819 | -0.013 | -0.006 | [-0.204, 0.177] | 0.890 |
| Parent smoking status: yes |  |  |  |  | 0.034 | 0.019 | [-0.133, 0.201] | 0.689 | 0.039 | 0.022 | [-0.127, 0.204] | 0.646 |
| Parental health literacy score |  |  |  |  |  |  |  |  | **0.012** | **0.145** | **[0.004, 0.020]** | **0.002** |
| ***R²*** | **0.187** | | | | **0.206** | | | | **0.224** | | | |
| ***ΔR²*** | **0.187** | | | | **0.018** | | | | **0.019** | | | |
| **ΔF, (p)** | **23.190, p<.001** | | | | **0.806, p=0.634** | | | | **9.436, p=0.002** | | | |
| Dependent variable: nutrition subscale score; B, unstandardized regression coefficient; β, standardized regression coefficient; CI, confidence interval; R², coefficient of determination; ΔR², change in explained variance; ΔF, change in F statistic. Reference categories were male child, mother, absence of chronic disease, no regular medication use, no smoking, primary education, and moderate economic status. Durbin-Watson: 1.901. Bold values indicate p<.05. | | | | | | | | | | | | |

**Supplementary Table S4.** Hierarchical multiple linear regression models for the sleep subscale score

|  | **Model 1: Child-related characteristics** | | | | **Model 2: + Parent and family characteristics** | | | | **Model 3: + Parental health literacy** | | | |
| --- | --- | --- | --- | --- | --- | --- | --- | --- | --- | --- | --- | --- |
| **Independent variables** | **B** | **β** | **95% CI for B** | **p** | **B** | **β** | **95% CI for B** | **p** | **B** | **β** | **95% CI for B** | **p** |
| *(Constant)* | 1.680 | - | [0.744, 2.617] | <0.001 | 2.334 | - | [1.189, 3.479] | <0.001 | 2.233 | - | [1.052, 3.413] | <0.001 |
| Child sex: female | -0.111 | -0.063 | [-0.280, 0.058] | 0.195 | -0.118 | -0.067 | [-0.289, 0.053] | 0.177 | -0.115 | -0.065 | [-0.287, 0.056] | 0.188 |
| Child age | **0.435** | **0.246** | **[0.266, 0.604]** | **<0.001** | **0.407** | **0.230** | **[0.233, 0.581]** | **<0.001** | **0.395** | **0.223** | **[0.217, 0.572]** | **<0.001** |
| Chronic disease in the child: yes | -0.148 | -0.041 | [-0.624, 0.328] | 0.542 | -0.160 | -0.044 | [-0.638, 0.318] | 0.512 | -0.147 | -0.041 | [-0.626, 0.333] | 0.549 |
| Regular medication use by the child: yes | -0.376 | -0.092 | [-0.907, 0.155] | 0.165 | -0.308 | -0.076 | [-0.843, 0.228] | 0.259 | -0.306 | -0.075 | [-0.842, 0.230] | 0.262 |
| Responding parent: father |  |  |  |  | 0.099 | 0.045 | [-0.141, 0.339] | 0.419 | 0.093 | 0.042 | [-0.148, 0.334] | 0.450 |
| Parent age |  |  |  |  | -0.009 | -0.049 | [-0.028, 0.011] | 0.375 | -0.008 | -0.046 | [-0.027, 0.011] | 0.413 |
| Parent education: high school |  |  |  |  | -0.259 | -0.130 | [-0.528, 0.010] | 0.059 | -0.270 | -0.136 | [-0.540, 0.001] | 0.051 |
| Parent education: associate degree |  |  |  |  | **-0.313** | **-0.136** | **[-0.607, -0.019]** | **0.037** | **-0.333** | **-0.144** | **[-0.632, -0.034]** | **0.029** |
| Parent education: bachelor's degree |  |  |  |  | **-0.288** | **-0.153** | **[-0.566, -0.010]** | **0.042** | **-0.301** | **-0.159** | **[-0.581, -0.021]** | **0.035** |
| Parent education: master's or doctoral degree |  |  |  |  | -0.390 | -0.116 | [-0.797, 0.016] | 0.060 | -0.401 | -0.119 | [-0.809, 0.007] | 0.054 |
| Perceived economic status: low |  |  |  |  | 0.025 | 0.006 | [-0.388, 0.437] | 0.906 | 0.029 | 0.007 | [-0.384, 0.442] | 0.889 |
| Perceived economic status: good |  |  |  |  | 0.283 | 0.069 | [-0.109, 0.674] | 0.157 | 0.291 | 0.072 | [-0.101, 0.684] | 0.145 |
| Number of children |  |  |  |  | 0.002 | 0.002 | [-0.117, 0.122] | 0.972 | 0.005 | 0.005 | [-0.115, 0.125] | 0.934 |
| Chronic disease in the parent: yes |  |  |  |  | -0.150 | -0.054 | [-0.417, 0.117] | 0.271 | -0.147 | -0.053 | [-0.414, 0.121] | 0.281 |
| Parent smoking status: yes |  |  |  |  | 0.168 | 0.073 | [-0.063, 0.400] | 0.154 | 0.170 | 0.073 | [-0.062, 0.402] | 0.151 |
| Parental health literacy score |  |  |  |  |  |  |  |  | 0.004 | 0.036 | [-0.007, 0.015] | 0.481 |
| ***R²*** | **0.075** | | | | **0.105** | | | | **0.106** | | | |
| ***ΔR²*** | **0.075** | | | | **0.030** | | | | **0.001** | | | |
| **ΔF, (p)** | **8.186, p<.001** | | | | **1.186, p=0.294** | | | | **0.498, p=0.481** | | | |
| Dependent variable: sleep subscale score; B, unstandardized regression coefficient; β, standardized regression coefficient; CI, confidence interval; R², coefficient of determination; ΔR², change in explained variance; ΔF, change in F statistic. Reference categories were male child, mother, absence of chronic disease, no regular medication use, no smoking, primary education, and moderate economic status. Durbin-Watson: 2.046. Bold values indicate p<.05. | | | | | | | | | | | | |

**Supplementary Table S5.** Hierarchical multiple linear regression models for the mental health and social relationships subscale score

|  | **Model 1: Child-related characteristics** | | | | **Model 2: + Parent and family characteristics** | | | | **Model 3: + Parental health literacy** | | | |
| --- | --- | --- | --- | --- | --- | --- | --- | --- | --- | --- | --- | --- |
| **Independent variables** | **B** | **β** | **95% CI for B** | **p** | **B** | **β** | **95% CI for B** | **p** | **B** | **β** | **95% CI for B** | **p** |
| *(Constant)* | 1.399 | - | [0.902, 1.896] | <0.001 | 1.685 | - | [1.074, 2.296] | <0.001 | 1.337 | - | [0.724, 1.951] | <0.001 |
| Child sex: female | -0.016 | -0.015 | [-0.105, 0.074] | 0.729 | -0.016 | -0.016 | [-0.108, 0.075] | 0.728 | -0.007 | -0.007 | [-0.096, 0.082] | 0.875 |
| Child age | **0.513** | **0.493** | **[0.424, 0.603]** | **<0.001** | **0.506** | **0.486** | **[0.413, 0.599]** | **<0.001** | **0.463** | **0.445** | **[0.371, 0.555]** | **<0.001** |
| Chronic disease in the child: yes | -0.118 | -0.056 | [-0.370, 0.135] | 0.361 | -0.143 | -0.068 | [-0.398, 0.112] | 0.271 | -0.098 | -0.046 | [-0.347, 0.152] | 0.441 |
| Regular medication use by the child: yes | -0.113 | -0.047 | [-0.395, 0.169] | 0.431 | -0.116 | -0.049 | [-0.402, 0.170] | 0.424 | -0.111 | -0.046 | [-0.389, 0.168] | 0.436 |
| Responding parent: father |  |  |  |  | -0.007 | -0.006 | [-0.136, 0.121] | 0.909 | -0.029 | -0.022 | [-0.154, 0.097] | 0.653 |
| Parent age |  |  |  |  | -0.006 | -0.057 | [-0.016, 0.004] | 0.259 | -0.004 | -0.036 | [-0.014, 0.006] | 0.465 |
| Parent education: high school |  |  |  |  | -0.069 | -0.059 | [-0.213, 0.074] | 0.343 | -0.106 | -0.090 | [-0.247, 0.035] | 0.141 |
| Parent education: associate degree |  |  |  |  | -0.038 | -0.028 | [-0.195, 0.119] | 0.633 | -0.107 | -0.079 | [-0.263, 0.049] | 0.178 |
| Parent education: bachelor's degree |  |  |  |  | -0.035 | -0.031 | [-0.183, 0.114] | 0.646 | -0.078 | -0.071 | [-0.224, 0.067] | 0.291 |
| Parent education: master's or doctoral degree |  |  |  |  | -0.152 | -0.077 | [-0.369, 0.065] | 0.170 | -0.187 | -0.095 | [-0.399, 0.025] | 0.083 |
| Perceived economic status: low |  |  |  |  | 0.075 | 0.030 | [-0.145, 0.295] | 0.502 | 0.091 | 0.036 | [-0.124, 0.306] | 0.405 |
| Perceived economic status: good |  |  |  |  | 0.076 | 0.032 | [-0.133, 0.285] | 0.475 | 0.106 | 0.044 | [-0.098, 0.310] | 0.308 |
| Number of children |  |  |  |  | -0.009 | -0.014 | [-0.073, 0.055] | 0.784 | 0.001 | 0.002 | [-0.061, 0.064] | 0.967 |
| Chronic disease in the parent: yes |  |  |  |  | 0.092 | 0.056 | [-0.051, 0.234] | 0.208 | 0.101 | 0.062 | [-0.038, 0.240] | 0.152 |
| Parent smoking status: yes |  |  |  |  | 0.078 | 0.057 | [-0.046, 0.201] | 0.218 | 0.083 | 0.061 | [-0.038, 0.203] | 0.178 |
| Parental health literacy score |  |  |  |  |  |  |  |  | **0.014** | **0.207** | **[0.008, 0.019]** | **<0.001** |
| ***R²*** | **0.246** | | | | **0.262** | | | | **0.301** | | | |
| ***ΔR²*** | **0.246** | | | | **0.016** | | | | **0.039** | | | |
| **ΔF, (p)** | **32.829, p<.001** | | | | **0.761, p=0.679** | | | | **21.505, p<.001** | | | |
| Dependent variable: mental health and social relationships subscale score; B, unstandardized regression coefficient; β, standardized regression coefficient; CI, confidence interval; R², coefficient of determination; ΔR², change in explained variance; ΔF, change in F statistic. Reference categories were male child, mother, absence of chronic disease, no regular medication use, no smoking, primary education, and moderate economic status. Durbin-Watson: 1.850. Bold values indicate p<.05. | | | | | | | | | | | | |

**Supplementary Table S6.** Hierarchical multiple linear regression models for the neglect and abuse subscale score

|  | **Model 1: Child-related characteristics** | | | | **Model 2: + Parent and family characteristics** | | | | **Model 3: + Parental health literacy** | | | |
| --- | --- | --- | --- | --- | --- | --- | --- | --- | --- | --- | --- | --- |
| **Independent variables** | **B** | **β** | **95% CI for B** | **p** | **B** | **β** | **95% CI for B** | **p** | **B** | **β** | **95% CI for B** | **p** |
| *(Constant)* | 1.501 | - | [1.001, 2.001] | <0.001 | 1.851 | - | [1.249, 2.453] | <0.001 | 1.460 | - | [0.861, 2.060] | <0.001 |
| Child sex: female | -0.018 | -0.017 | [-0.108, 0.073] | 0.702 | -0.027 | -0.026 | [-0.117, 0.063] | 0.561 | -0.016 | -0.016 | [-0.104, 0.071] | 0.711 |
| Child age | **0.501** | **0.483** | **[0.411, 0.591]** | **<0.001** | **0.497** | **0.480** | **[0.406, 0.589]** | **<0.001** | **0.449** | **0.433** | **[0.359, 0.539]** | **<0.001** |
| Chronic disease in the child: yes | -0.096 | -0.045 | [-0.350, 0.158] | 0.459 | -0.116 | -0.055 | [-0.368, 0.135] | 0.363 | -0.066 | -0.031 | [-0.310, 0.178] | 0.596 |
| Regular medication use by the child: yes | -0.038 | -0.016 | [-0.321, 0.246] | 0.794 | -0.030 | -0.013 | [-0.312, 0.251] | 0.832 | -0.024 | -0.010 | [-0.296, 0.248] | 0.863 |
| Responding parent: father |  |  |  |  | 0.013 | 0.010 | [-0.113, 0.140] | 0.835 | -0.010 | -0.008 | [-0.133, 0.112] | 0.868 |
| Parent age |  |  |  |  | -0.004 | -0.041 | [-0.014, 0.006] | 0.409 | -0.002 | -0.018 | [-0.012, 0.008] | 0.715 |
| Parent education: high school |  |  |  |  | -0.011 | -0.010 | [-0.153, 0.130] | 0.876 | -0.052 | -0.045 | [-0.190, 0.085] | 0.457 |
| Parent education: associate degree |  |  |  |  | -0.006 | -0.004 | [-0.160, 0.149] | 0.940 | -0.083 | -0.061 | [-0.235, 0.069] | 0.283 |
| Parent education: bachelor's degree |  |  |  |  | -0.014 | -0.012 | [-0.160, 0.132] | 0.852 | -0.063 | -0.057 | [-0.205, 0.080] | 0.386 |
| Parent education: master's or doctoral degree |  |  |  |  | **-0.224** | **-0.114** | **[-0.438, -0.010]** | **0.040** | **-0.264** | **-0.134** | **[-0.471, -0.057]** | **0.013** |
| Perceived economic status: low |  |  |  |  | -0.197 | -0.078 | [-0.413, 0.020] | 0.075 | -0.179 | -0.071 | [-0.389, 0.031] | 0.094 |
| Perceived economic status: good |  |  |  |  | 0.086 | 0.036 | [-0.120, 0.292] | 0.410 | 0.120 | 0.050 | [-0.079, 0.319] | 0.238 |
| Number of children |  |  |  |  | **-0.085** | **-0.130** | **[-0.148, -0.022]** | **0.008** | **-0.074** | **-0.113** | **[-0.135, -0.013]** | **0.018** |
| Chronic disease in the parent: yes |  |  |  |  | 0.035 | 0.022 | [-0.105, 0.175] | 0.623 | 0.046 | 0.028 | [-0.090, 0.182] | 0.504 |
| Parent smoking status: yes |  |  |  |  | **0.135** | **0.100** | **[0.014, 0.257]** | **0.029** | **0.141** | **0.104** | **[0.023, 0.259]** | **0.019** |
| Parental health literacy score |  |  |  |  |  |  |  |  | **0.015** | **0.234** | **[0.010, 0.021]** | **<0.001** |
| ***R²*** | **0.232** | | | | **0.279** | | | | **0.328** | | | |
| ***ΔR²*** | **0.232** | | | | **0.047** | | | | **0.049** | | | |
| **ΔF, p** | **30.327, p<.001** | | | | **2.316, p=0.009** | | | | **28.409, p<.001** | | | |
| Dependent variable: neglect and abuse subscale score; B, unstandardized regression coefficient; β, standardized regression coefficient; CI, confidence interval; R², coefficient of determination; ΔR², change in explained variance; ΔF, change in F statistic. Reference categories were male child, mother, absence of chronic disease, no regular medication use, no smoking, primary education, and moderate economic status. Durbin-Watson: 1.784. Bold values indicate p<.05. | | | | | | | | | | | | |
